# Care of the Skin

**Published:** 1890-10

**Authors:** 


### CARE OF THE SKIN.

It is common to speak as if any care of the skin beyond personal cleanliness was foolish and a sinful waste of time. This is but a remnant of the old idea, which rigid Puritans held in common with Catholic ascetics, that it was inducive to a saintly frame of mind to make the dress as hideous as possible, and show one's contempt for the natural beauty which God has lavished all over the face of the earth. A soft, beautiful complexion is certainly an attraction which every woman should desire, and any simple means, which does not occupy time needed for more important matters, should be tried to attain such an end. There are many complexions which chafe readily, and tan in the spring winds. A simple preparation of sweet cream rubbed into the skin after washing it thoroughly, is a remedy for this trouble. This should be applied at night, just before retiring, and the next morning the face should be washed thoroughly, first in lukewarm water, and afterwards in cold, to give tone to the muscles. Some ladies who do not find glycerine irritating to the skin, use in the same way a small portion of it diluted with half its bulk of rose-water. This preparation is rubbed in the face, and hands, and gloves are worn at night. A little ammonia in the water is a help toward keeping the skin firm, and free from wrinkles. There certainly is no remedy for wrinkles after they come. It should be remembered,however, that an amiable temper, a clear conscience, and freedom from a disposition to worry over the petty annoyances of life are qualities of mind and heart that will keep the face free from wrinkles and beautiful to the ripest old age. A habit common with studious children, and those who are near-sighted, is to knit the brow. This often causes premature lengthwise lines in the forehead.

### ESSENTIAL OILS.

The composition of all essential oils is one and the same. There is no chemical difference between them. Those of bergamot, birch, chamomile, caraway, hops, juniper, lemon, myrtle, nutmeg, orange, parsley, pepper, savin, thyme, tolu, and valerian, all have the same composition, expressed by the empirical symbol $C_{10}H_{16}$. The widely differing properties of these oils are explained by chemists as due to the different structure, or way in which the atoms are arranged in the molecule, just as the same number of bricks may be arranged to form a house, church, store, or many other different buildings.

